# Current Status and Future Aspects of Gadolinium Oxide Nanoparticles as Positive Magnetic Resonance Imaging Contrast Agents

**DOI:** 10.3390/nano15171340

**Published:** 2025-09-01

**Authors:** Endale Mulugeta, Tirusew Tegafaw, Ying Liu, Dejun Zhao, Xiaoran Chen, Ahrum Baek, Jihyun Kim, Yongmin Chang, Gang Ho Lee

**Affiliations:** 1 Department of Chemistry, College of Natural Sciences, Kyungpook National University, Taegu 41566, Republic of Korea; endexindex05@gmail.com (E.M.); tirukorea@gmail.com (T.T.); ly1124161@gmail.com (Y.L.); djzhao.chem@gmail.com (D.Z.); tsukiyovo@gmail.com (X.C.); 2 Institute of Biomedical Engineering Research, Kyungpook National University, Taegu 41944, Republic of Korea; baxun@naver.com; 3 Department of Chemistry Education, Teachers’ College, Kyungpook National University, Taegu 41566, Republic of Korea; jkim23@knu.ac.kr; 4 Department of Molecular Medicine, School of Medicine, Kyungpook National University, Taegu 41944, Republic of Korea

**Keywords:** gadolinium oxide nanoparticle, surface modification, MRI, contrast agent, future aspects

## Abstract

Although numerous studies have investigated gadolinium oxide (Gd_2_O_3_) nanoparticles (NPs) as positive (T_1_) magnetic resonance imaging (MRI) contrast agents (CAs), comprehensive reviews on this topic remain scarce. Therefore, it is essential to evaluate their current status and outline prospects. Despite promising physicochemical properties such as considerably higher relaxivities compared to 3–5 s^−1^mM^−1^ of clinically approved Gd(III)-chelate contrast agents and encouraging results from in vivo animal studies such as highly improved contrast enhancements, drug loading, and tumor targeting, extensive in vivo toxicity assessments including long-term toxicity and formulation advancements suitable for renal excretion (d < ~3 nm) are still required for clinical translation. This review summarizes the synthesis, characterization, in vitro and in vivo toxicity, and in vivo MRI applications of surface-modified Gd_2_O_3_ NPs as T_1_ MRI CAs. Finally, future perspectives on the development of surface-modified Gd_2_O_3_ NPs as potential next-generation T_1_ MRI CAs are discussed.

## 1. Introduction

Molecular imaging plays a critical role in medical treatments by providing detailed anatomical information through high-resolution imaging [1,2]. Various imaging modalities have been developed, including fluorescence imaging (FI), magnetic resonance imaging (MRI), X-ray computed tomography (CT), positron emission tomography (PET), ultrasound imaging (USI), and single-photon emission computed tomography (SPECT) [3,4]. These techniques enable the detection, diagnosis, and monitoring of diseases. Among them, MRI is particularly valued for its non-invasive nature as it employs non-ionizing radiofrequency radiation and allows for whole-body imaging with unlimited penetration depth in soft tissues, making it a widely adopted modality [5]. However, MRI suffers from relatively low sensitivity due to the small population difference between the two proton spin states [6,7], which limits its ability to detect small or early-stage lesions. This limitation can be mitigated by the use of contrast agents (CAs), which enhance signal intensity by accelerating proton spin relaxation processes, thereby improving detection sensitivity [8,9].

Various types of MRI CAs have been investigated, including Gd(III)- and Mn(II)-chelates [10,11], Gd- [12] and Mn-containing nanoparticles (NPs) [13] for positive (T_1_) MRI, and superparamagnetic iron oxide nanoparticles (SPIONs) [14,15] for negative (T_2_) MRI. Currently, only Gd(III)-chelates are approved for clinical use as T_1_ MRI CAs [16,17]. Although SPIONs were previously commercialized, their use is now minimal [18]. Gd^3+^, with seven unpaired 4f-electrons and a high spin magnetic moment (s = 7/2), is ideal for use in T_1_ MRI CAs [19]. Consequently, Gd_2_O_3_ NPs can induce stronger T_1_ proton spin relaxations than Mn-and Fe-based NPs owing to lower electron spin magnetic moments of Mn^2+^ (s = 5/2) and Fe^3+^ (s = 5/2) than Gd^3+^ [16,20,21,22,23,24,25]. For example, this can be noticed from the r_1_ value (33.4 s^−1^mM^−1^) of free Gd^3+^ ions-grafted NDs [26], which is higher than that (13.5 s^−1^mM^−1^) of free Mn^2+^ ions-grafted NDs [27]. However, after PVP coating, r_1_ value of free Gd^3+^ ions-grafted NDs dropped to 15.9 s^−1^mM^−1^ [28]. T_1_ MRI CAs are generally preferred over T_2_ MRI CAs because signal enhancement is easier to detect than signal loss [29]. Moreover, SPIONs can cause signal darkening and susceptibility-induced artifacts, which may complicate image interpretation [30,31,32,33]. In contrast, Gd_2_O_3_ NPs produce the high-quality positive contrast images with less artificial signal voids, thereby offering clearer diagnostic outcomes [34].

However, Gd^3+^ ions are inherently toxic and non-biodegradable through metabolic processes [35]. To mitigate this toxicity, they are commonly administered as Gd(III)-chelates [35,36,37,38,39]. Although Gd(III)-chelates effectively enhance positive contrasts in MRI, they exhibit low sensitivity due to their low longitudinal proton spin relaxivity (r_1_) values and lack of specificity. Moreover, they have been associated with serious adverse effects, including nephrogenic systemic fibrosis (NSF) in patients with renal impairment, a condition characterized by skin and organ thickening and darkening, ultimately impairing organ function [40,41,42]. Recent studies have also indicated that repeated administration of Gd(III)-chelates can lead to gadolinium deposition in the brain, potentially causing neurotoxicity [43,44].

With advancements in nanotechnology, Gd_2_O_3_ NPs have emerged as promising alternatives to Gd(III)-chelates, offering several advantages. These NPs exhibit a high density of Gd^3+^ ions per NP, resulting in higher r_1_ values [45,46,47,48], compared to 3–5 s^−1^mM^−1^ [17,38] of Gd(III)-chelates. They also demonstrate prolonged blood circulation times due to the enhanced permeability and retention (EPR) effect [46], enabling extended imaging and diagnosing windows [47]. Furthermore, their high surface-to-volume ratios facilitate drug loading and conjugation of targeting ligands, making them suitable for theranostic applications [48,49,50]. However, their drawback is potential toxicity, which needs to be clearly and thoroughly investigated for clinical translations. For biomedical applications, Gd_2_O_3_ NPs must be surface-modified with hydrophilic and biocompatible ligands to ensure colloidal stability, biocompatibility, and functionalization potential. Various synthesis methods have been reported, including polyol [51,52], dimethyl sulfoxide (DMSO) [53,54], thermal decomposition [55], and hydrothermal methods [56]. Surface modifications have employed a range of biocompatible and hydrophilic ligands such as polyacrylic acid (PAA) [51,57], poly(methyl vinyl ether-alt-maleic acid) (PMVEMA) [58,59,60], poly(acrylic acid-co-maleic acid) (PAAMA) [61,62], polyaspartic acid (PASA) [63], D-glucuronic acid [64], citric acid (CA) [65], dextran [66], polyethylene glycol (PEG) [67], or polyvinylpyrrolidone (PVP) [68]. These ligand coatings are critical not only for stability and functionality but also for reducing toxicity by preventing the release of free Gd^3+^ ions from the NPs.

**Figure 1:**
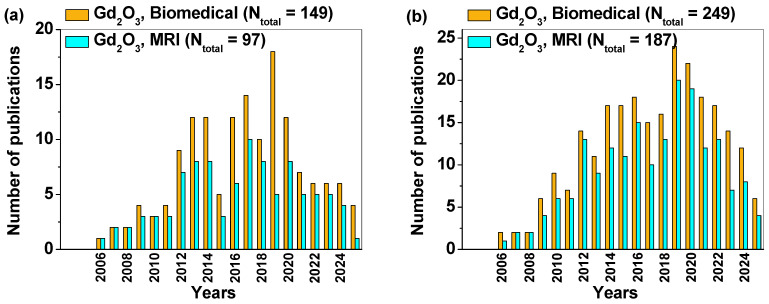
Number of publications on Gd_2_O_3_ NPs related to biomedical applications and T_1_ MRI CAs, based on data from (**a**) Scopus and (**b**) Web of Science, up to 20 June 2025.

To date, few comprehensive reviews have focused specifically on surface-coated Gd_2_O_3_ NPs as T_1_ MRI CAs. However, as illustrated in Figure 1, a substantial number of research articles have been reported on surface-coated Gd_2_O_3_ NPs as T_1_ MRI CAs and their biomedical applications. Therefore, it is both timely and necessary to evaluate the current status of this research and to discuss future directions. As shown in Figure 1, the annual growth rate of publications increased up to 2019 and then decreased afterwards. This trend reflects the increase in the development of various types of gadolinium NP formulations, in vivo MRI, and biotoxicity studies up to 2019, and then the decrease afterwards. This review covers key aspects, including synthesis methods, physicochemical characterization, in vitro and in vivo toxicity assessments, and in vivo MRI applications. Finally, future perspectives for clinical translation of surface-coated Gd_2_O_3_ NPs are outlined.

## 2. Synthesis of Surface-Modified Gd_2_O_3_ NPs

To date, various synthetic approaches have been employed to prepare surface-modified Gd_2_O_3_ NPs for biomedical applications. These include polyol [51,52], DMSO [53], thermal decomposition [55], and hydrothermal methods [56]. As summarized in Table 1, each method presents distinct advantages and limitations in terms of synthesis complexity, scalability, and environmental impact.

### 2.1. Polyol Method

**Figure 2:**
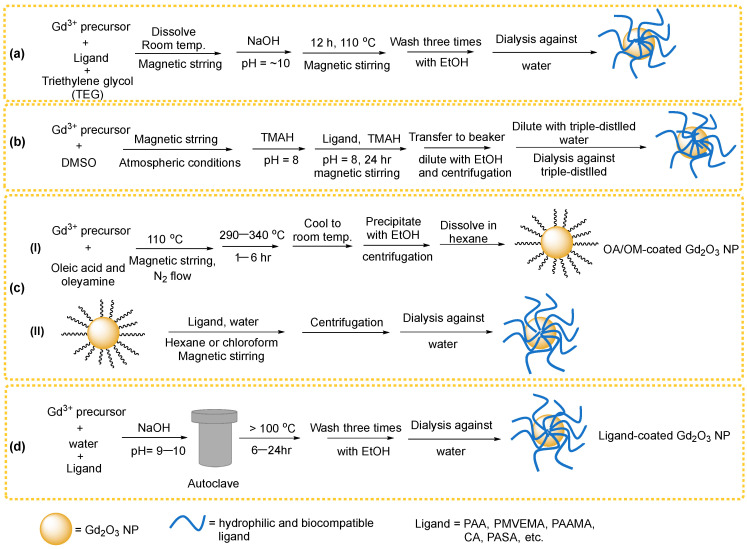
Synthesis schemes for hydrophilic and biocompatible ligand-coated Gd_2_O_3_ NPs: (**a**) one-pot polyol method, (**b**) one-pot DMSO method, (**c**) (**I**) thermal decomposition method yielding OA/OM-coated Gd_2_O_3_ NPs and (**II**) subsequent ligand exchange with hydrophilic and biocompatible ligands, and (**d**) one-pot hydrothermal method.

The polyol method is effective for synthesizing ultrasmall Gd_2_O_3_ NPs, with average particle diameters of approximately 2.0 nm, via a one-pot process that simultaneously enables surface modification with hydrophilic and biocompatible ligands [34,51,58,61,63,64,69,70,71,72,73,74,75]. High boiling point polyols, such as ethylene glycol (EG), diethylene glycol (DEG), triethylene glycol (TEG), and polyethylene glycol (PEG), are commonly used as solvents. These polyols also function as stabilizing agents, preventing particle aggregation. A general scheme for the one-pot polyol synthesis of surface-coated Gd_2_O_3_ NPs is displayed in Figure 2a. The process involves a Gd^3+^ precursor, surface-coating ligands, NaOH, and a polyol solvent, all reacted under atmospheric conditions with magnetic stirring. Initially, the Gd^3+^ precursor solution is prepared by dissolving Gd^3+^ salt, such as GdCl_3_·xH_2_O, and ligands such as PAA in a polyol such as TEG within a three-necked round-bottom flask. This mixture is stirred magnetically at room temperature under atmospheric conditions until complete dissolution. Separately, a NaOH solution is prepared by dissolving NaOH in TEG in a beaker and is then slowly added to the precursor solution until the pH reaches 8–10. The resulting mixture is stirred at ~110 °C for ~12 h to yield ligand-coated Gd_2_O_3_ NPs.

For example, Lee’s group synthesized ultrasmall Gd_2_O_3_ NPs using various hydrophilic ligands, such as PAA, PMVEMA, PAAMA, PASA, CA, and D-glucuronic acid, via the one-pot polyol method [51,58,61,63,64]. In another study, Guleria et al. synthesized Gd_2_O_3_ NPs using polyols of varying chain lengths, including DEG, TEG, tetraethylene glycol (TeEG), and PEG-200, and observed a linear correlation between the glycol chain length and the resulting NP size [70].

### 2.2. DMSO Method

The DMSO method is suitable for low-temperature synthesis (<60 °C), enabling the production of ultrasmall Gd_2_O_3_ NPs with uniform particle size and simultaneous one-pot surface modification using hydrophilic and biocompatible ligands [53,76,77]. As shown in Figure 2b, Gd^3+^ precursor such as Gd(acetate)_3_·xH_2_O is dissolved in DMSO under atmospheric conditions with magnetic stirring until a transparent solution is obtained. Tetramethylammonium hydroxide (TMAH) solution is then added dropwise to adjust the pH to ~8, resulting in a cloudy solution. This mixture is stirred magnetically for 24 h to allow the formation of ultrasmall Gd_2_O_3_ NPs. For surface coating, a hydrophilic ligand such as PAA is added to the reaction mixture, followed by the addition of TMAH to maintain pH at ~8. The solution is stirred for another 24 h to ensure uniform ligand surface coating. The resulting ligand-coated Gd_2_O_3_ NPs are purified by repeated ethanol washing and centrifugation to remove unreacted reagents and solvents. Finally, the surface-coated NPs are dispersed in triple-distilled water and subjected to dialysis against water to eliminate residual impurities.

For example, Uvdal and co-workers synthesized Gd_2_O_3_ NPs (d = 4–5 nm) by dissolving Gd(acetate)_3_·xH_2_O in DMSO and adding TMAH in ethanol under ambient conditions with magnetic stirring [53]. In another example, Cui et al. synthesized Gd_2_O_3_ NPs using Gd(acetate)_3_·xH_2_O and TMAH in DMSO at room temperature and subsequently conjugated the NPs with carboxyfluorescein [FI]-polyethylene glycol [PEG]-bombesin (BBN), resulting in NPs with a particle diameter of 52.3 nm [77].

### 2.3. Thermal Decomposition

The thermal decomposition method yields monodispersed Gd_2_O_3_ NPs with controlled particle size; however, it requires high temperatures, organic solvents such as OA and OM, and an inert gas atmosphere, rendering the synthesis costly and environmentally unfriendly [55,78,79,80,81,82]. Additionally, this method does not permit one-pot synthesis for surface modification. As shown in Figure 2(cI), Gd^3+^ precursor is added to a mixture of OA and OM in a three-neck round-bottom flask and heated to ~110 °C under a flow of argon or nitrogen to remove moisture and dissolved gases. The reaction mixture is then refluxed at 290–340 °C for 1–6 h. After cooling to room temperature, the Gd_2_O_3_ NPs are precipitated by adding ethanol, collected by centrifugation, and redispersed in toluene: this washing procedure is repeated three times. The purified NPs are subsequently dispersed and stored in hexane or chloroform. For surface coating, the hydrophobic ligands on the NP surfaces, primarily OA, with a minor contribution from OM due to the stronger binding affinity of the -COOH group than the -NH_2_ group, are replaced with hydrophilic ligands such as polyvinyl pyrrolidone (PVP) through ligand exchange (Figure 2(cII)). The resulting hydrophilic ligand-coated Gd_2_O_3_ NPs are dispersed in water and further purified through dialysis against water.

For example, Fang et al. synthesized monodispersed ultrasmall Gd_2_O_3_ NPs (d = 2.9 nm) capped with hydrophobic OA, via thermal decomposition of a Gd(OA)_3_ precursor in a mixture of OA and OM, which acted as both solvent and surfactant [78]. The OA-capped Gd_2_O_3_ NPs were subsequently converted to PVP-Gd_2_O_3_ NPs through ligand exchange with PVP for use as T_1_ MRI CAs. Similarly, Cai et al. prepared OA/OM-capped Gd_2_O_3_ nanoplates (length × thickness = 10 × 1 nm) via thermal decomposition of Gd(OA)_3_ at 340 °C using OA and OM as solvent and capping agents. These nanoplates were encapsulated with *N*-dodecyl-polyethylene imine (PEI)-PEG polymers to form hydrophilic nanoplate clusters (d = 95 nm) for application as T_1_ MRI CAs [55].

### 2.4. Hydrothermal Method

The hydrothermal method is considered environmentally friendly as it utilizes water as the solvent. The synthesis is controlled by adjusting temperature and pressure [46,56,83,84,85,86]. As illustrated in Figure 2d, the Gd^3+^ precursor is dissolved in deionized water. Separately, a hydrophilic ligand is dissolved in deionized water at room temperature and added to the Gd^3+^ solution under magnetic stirring. An aqueous solution of NaOH or urea is then added dropwise under magnetic stirring to adjust the pH to 9–10. The resulting solution is transferred into an autoclave and subjected to hydrothermal treatment at elevated temperatures (typically above 150 °C) for 6–24 h. The final precipitates are washed three times with ethanol, dispersed in triple-distilled water, and further purified via dialysis against water.

For example, Wu et al. synthesized hyaluronic acid (HA)-coated Gd_2_O_3_ NPs (d = 105 nm) using the hydrothermal method [46]. In this procedure, HA was dissolved in water under vigorous magnetic stirring at ambient temperature overnight. GdCl_3_·6H_2_O and NaOH were then added, followed by magnetic stirring for 5 min to form a homogeneous clear solution. The mixture was transferred to an autoclave, sealed, and heated at 120 °C for 6 h. The resulting HA-coated Gd_2_O_3_ NPs were purified through dialysis against water to remove impurities.

## 3. Various Characterizations

Following synthesis, various characterization techniques can be employed to determine the physicochemical properties of surface-modified Gd_2_O_3_ NPs, as summarized in Table 2 [51,53,61,87]. These include high-resolution transmission electron microscopy (HRTEM) to determine particle size and morphology; dynamic light scattering (DLS) to measure hydrodynamic diameter; X-ray diffraction (XRD) to determine the crystal structure; Fourier transform-infrared (FT-IR) absorption spectroscopy to analyze surface coating; and thermogravimetric analysis (TGA) to estimate the surface-coating content. Additional methods include zeta potential measurements to assess the surface charge; inductively coupled plasma-atomic emission spectroscopy (ICP-AES) to quantify Gd concentration in solution; and vibrating sample magnetometry (VSM) or magnetic property measurement system (MPMS) to measure magnetic properties. MRI is used to determine the r_1_ and r_2_ relaxivities. In vitro cellular toxicity is assessed using standard assays such as methyl thiazolyl tetrazolium (MTT), cell counting kit (CCK)-8, and water-soluble tetrazolium (WST)-1 to evaluate cell viability. Collectively, these methods constitute a standard characterization protocol for surface-coated Gd_2_O_3_ NPs.

### 3.1. Particle Size and Morphology

Particle size and morphology are critical parameters for biomedical applications. These features are typically characterized using HRTEM operated at acceleration voltages above 200 kV. For TEM imaging, NPs are dispersed onto a copper grid coated with a carbon film by drop-casting a solution sample prepared in water or a water/ethanol mixture. High-resolution imaging also enables measurement of lattice fringes, which can aid in NP identification. In addition, elemental mapping provides the spatial distribution of constituent elements, allowing identification of both the NPs and their surface-coating ligands.

**Figure 3:**
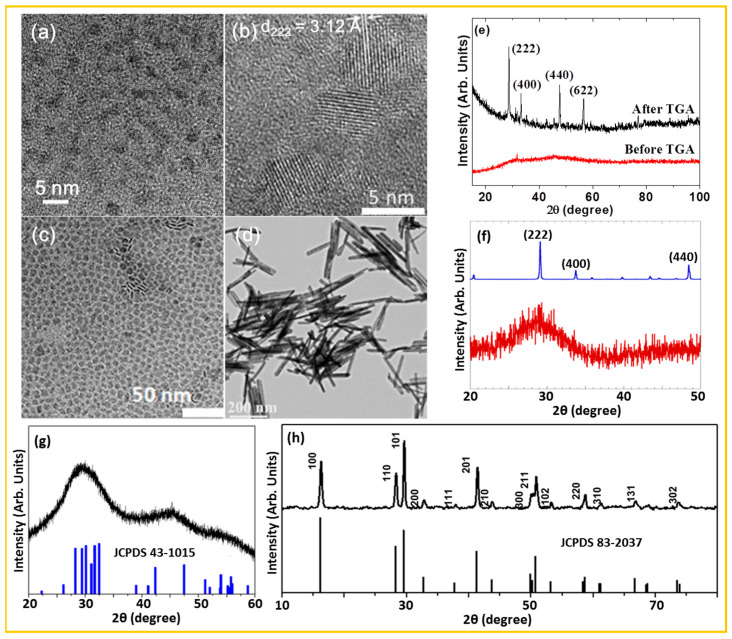
TEM images: (**a**) PAA-coated Gd_2_O_3_ NPs synthesized via the polyol method [51], (**b**) Gd_2_O_3_ NPs synthesized via the DMSO method [d_222_ indicates the lattice spacing between the (222) planes] [53], (**c**) OA/OM-capped Gd_2_O_3_ nanoplates synthesized via the thermal decomposition method [55], and (**d**) Gd(OH)_3_ nanorods synthesized via the hydrothermal method [56]. XRD patterns: (**e**) PAA-coated Gd_2_O_3_ NPs synthesized via the polyol method before (bottom spectrum) and after (top spectrum) TGA [51]. (**f**) Gd_2_O_3_ NPs synthesized via the DMSO method (bottom spectrum) and bulk Gd_2_O_3_ reference (top spectrum) [53]. (**g**) OA/OM-capped Gd_2_O_3_ nanoplates synthesized via the thermal decomposition method (top spectrum) and reference pattern with monoclinic structure (JCPDS No 43-1015) (bottom spectrum) [55]. (**h**) Gd(OH)_3_ nanorods synthesized via the hydrothermal method (top spectrum) and reference pattern with hexagonal structure (JCPDS No. 83-2037) (bottom spectrum) [56].

For example, Miao et al. synthesized PAA-coated ultrasmall Gd_2_O_3_ NPs (d_avg_ = 2.0 ± 0.1 nm) using a one-pot polyol method; the NPs remained colloidally stable in aqueous media without precipitation (Figure 3a) [51]. Ahrén et al. prepared Gd_2_O_3_ NPs with particle diameters of 4–5 nm via the DMSO method at room temperature (Figure 3b) [53]. These NPs exhibited good aqueous stability, with a zeta potential of +34.7 mV due to the presence of ammonium acetate on their surfaces. Cai et al. prepared OA/OM-capped Gd_2_O_3_ square nanoplates (thickness × length = 1 × 10 nm) via thermal decomposition of Gd(OA)_3_ at 340 °C, using OA and OM as both solvent and capping agent (Figure 3c) [55]. Yang et al. synthesized Gd(OH)_3_ nanorods by hydrothermal treatment of GdCl_3_ and triethylamine in water at 160 °C for 8 h in an autoclave [56]. Figure 3d displays the TEM image of the as-synthesized Gd(OH)_3_ nanorods (length × width = 200 × 10 nm).

### 3.2. Crystal Structure

XRD analysis provides critical information on the crystal structure of NPs and allows identification of the synthesized products. Powder samples are typically used, and the 2θ scan range is set between 10 and 100°. Diffraction peaks are indexed using Miller indices (hkl), and the unit cell parameters (a, b, c, α, β, γ) are calculated using Bragg’s law, in combination with interplanar spacing equations derived from the assigned crystal system. In addition, the average particle diameter can be roughly estimated using the Scherrer equation, which employs the full width at half maximum (FWHM) of selected XRD peaks [88]. Ultrasmall NPs (d < 3 nm) often exhibit poor crystallinity and may appear amorphous in XRD due to their limited long-range order. The pair distribution function analysis is recommended for ultrasmall NPs with amorphous XRD patterns because it offers the short-range local atomic structure of amorphous NPs [89]. However, heat treatment can induce particle growth and crystallization, allowing crystal structure determination [90].

For example, the XRD patterns corresponding to the gadolinium-based nanomaterials displayed in the TEM images in Figure 3a–d are provided in Figure 3e–h, respectively. The ultrasmall Gd_2_O_3_ NPs synthesized via the polyol and DMSO methods (TEM images in Figure 3a and Figure 3b, respectively) displayed broad, amorphous XRD patterns, indicating poor crystallinity (bottom spectra in Figure 3e,f) [51,53]. After thermal treatment, the NPs exhibited diffraction peaks corresponding to the cubic phase of Gd_2_O_3_ (top spectrum in Figure 3e) [51]. The top spectrum in Figure 3f represents the reference pattern of commercial bulk cubic Gd_2_O_3_ [53]. The Gd_2_O_3_ nanoplates synthesized via the thermal decomposition method (TEM image in Figure 3c), displayed broad peaks in the XRD pattern (top spectrum in Figure 3g) [55], which were indexed to a poorly developed monoclinic phase of Gd_2_O_3_ (JCPDS No 43-1015) [80,82,91], as shown in the reference pattern (bottom spectrum in Figure 3g). The XRD pattern of the Gd(OH)_3_ nanorods synthesized via the hydrothermal method (TEM image in Figure 3d) is presented in the top spectrum of Figure 3h and matches well with the reference pattern for the hexagonal phase of Gd(OH)_3_ (JCPDS No. 83-2037) (bottom spectrum in Figure 3h) [56].

### 3.3. Hydrodynamic Diameter

The hydrodynamic diameters (a_avg_) of NPs are measured using samples dispersed in water. The a_avg_ values are typically larger than the corresponding d_avg_ values due to the presence of surface-coating ligands and the hydration layer formed by adsorbed water molecules. The a_avg_ is influenced by particle size, morphology, and hydrophilicity of the surface ligands. Larger and more hydrophilic ligands attract more water molecules, thereby increasing the a_avg_ and enhancing colloidal stability. In particular, polymer ligands tend to attract more water molecules than small molecules, resulting in larger a_avg_ values and improved colloidal stability [92].

**Figure 4:**
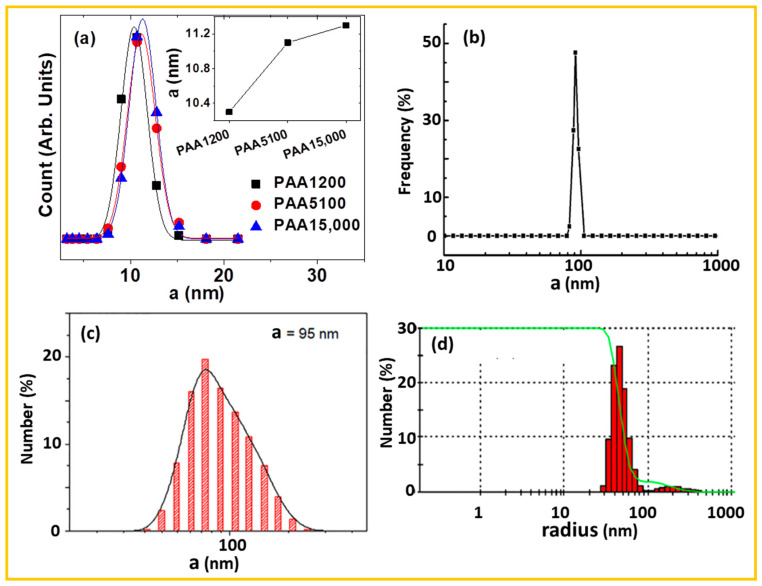
DLS patterns: (**a**) PAA-coated Gd_2_O_3_ NPs (PAA = 1200, 5100, and 15,000 Da) [75], (**b**) FI-PEG-BBN-coated Gd_2_O_3_ NPs [77], (**c**) Gd_2_O_3_@*N*-dodecyl-PEI-PEG nanoplate clusters [55], and (**d**) CA-coated hollow Gd_2_O_3_ NPs [93]. a = hydrodynamic diameter.

For example, Miao et al. synthesized ultrasmall Gd_2_O_3_ NPs (d = 2.0 nm) coated with PAA of varying molecular weight by the polyol method. They observed an increase in a_avg_ values (10.3 → 11.1 → 11.3 nm) with increasing PAA size (1200 → 5100 → 15,000 amu) (Figure 4a) [75]. Cui et al. synthesized Gd_2_O_3_ NPs (d = 52.3 nm) grafted with carboxyfluorescein (FI)-polyethylene glycol (PEG)-bombesin (BBN) via the DMSO method [77], and reported a large a_avg_ of 90.6 nm (Figure 4b), attributed to the combination of large particle size and bulky polymeric and biomolecular coating. Similarly, Cai et al. synthesized Gd_2_O_3_@*N*-dodecyl-PEI-PEG nanoplates (thickness × length = 1 × 10 nm), which exhibited a large a_avg_ value of 95 nm (Figure 4c) owing to the thick polymer coating [55]. Siribbal et al. synthesized CA-coated hollow Gd_2_O_3_ NPs (d = 100 nm) using a hydrothermal method [93], reporting a very large a_avg_ value of 233.7 nm, due to the large particle size and surface modification (Figure 4d).

### 3.4. Surface-Coating Analysis

The surface coating of surface-modified Gd_2_O_3_ NPs can be analyzed using FT-IR absorption spectroscopy and TGA [94,95]. Dried powder samples are used for both measurements to minimize the influence of water. Characteristic absorption peaks of surface-coating ligands in the FT-IR absorption spectra provide direct evidence of their presence on the Gd_2_O_3_ NP surfaces. Additionally, their coating amount can be estimated from TGA data, as most organic ligands undergo thermal decomposition below 450 °C via oxidation in a flow of hot air during TGA [96,97,98]. The coating amount is typically expressed in weight percent (wt.%), calculated from the mass loss observed in the TGA curve, excluding the initial mass loss between room temperature and ~100 °C due to desorption of water and air.

**Figure 5:**
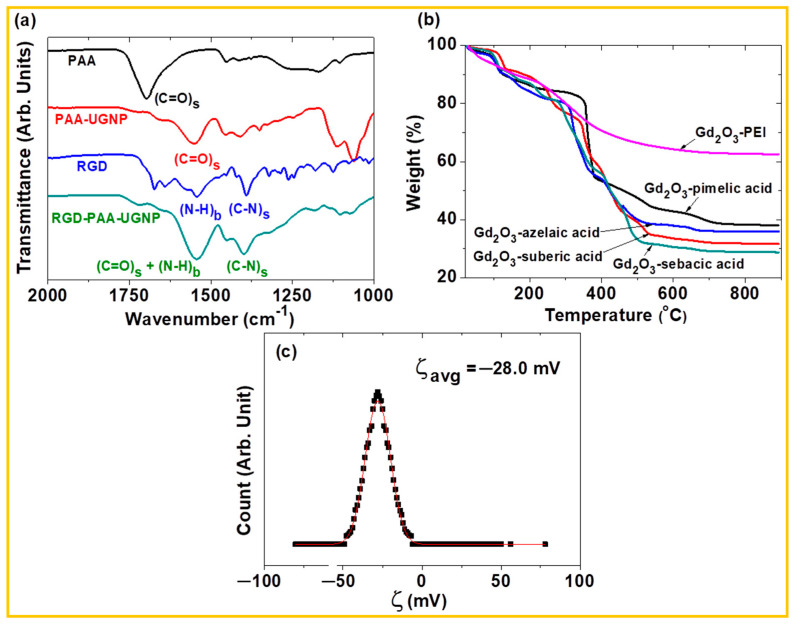
(**a**) FT-IR absorption spectra of PAA, PAA-UGNP, RGD, and RGD-PAA-UGNP (subscript labels “s” and “b” denote stretching and bending vibrations, respectively) [95]. (**b**) TGA curves of PEI, pimelic acid, azelaic acid, suberic acid, and sebacic acid coated-Gd_2_O_3_ NPs [99]. (**c**) Zeta potential (ζ) curve of PSA-coated Gd_2_O_3_ NPs [63].

For example, Ho et al. synthesized arginylglycylaspartic acid (RGD)-PAA-coated ultrasmall Gd_2_O_3_ NPs (denoted as RGD-PAA-UGNPs) for cancer imaging using in vivo T_1_ MRI in tumor-model mice [95]. They confirmed the successful coating of RGD-PAA onto UGNPs by recording FT-IR absorption spectra of PAA-UGNPs, RGD-PAA-UGNPs, free PAA, and free RGD (Figure 5a). The C=O stretching peak at 1553 cm^−1^ in both the PAA-UGNP and RGD–PAA–UGNP spectra confirmed the successful coordination of PAA ligands to the UGNP surfaces. This peak exhibited a red shift from that of free PAA at ~1700 cm^−1^ due to electrostatic interactions between the COO^−^ groups of PAA and surface Gd^3+^ ions of UGNP. Furthermore, the FT–IR absorption spectrum of RGD–PAA–UGNPs showed characteristic RGD peaks, including N–H bending at 1544 cm^−1^ and C–N stretching at 1390 cm^−1^, confirming successful conjugation of RGD to PAA through amide bond formation.

For surface-coating amount estimation, Park et al. measured TGA curves of ultrasmall Gd_2_O_3_ NPs coated with five different kinds of ligands: PEI, pimelic acid, azelaic acid, suberic acid, and sebacic acid (Figure 5b) [99]. The water and air desorption from the NPs occurred between room temperature and ~105 °C. Mass drops between ~105 and ~450 °C were attributed to the thermal decomposition or oxidative combustion of the coating ligands. The residual mass in TGA curves corresponded to Gd_2_O_3_ NPs. The measured surface-coating amounts were 52.3% for pimelic acid, 60.9% for suberic acid, 55.7% for azelaic acid, 63.1% for sebacic acid, and 30.5% for PEI-70000.

### 3.5. Zeta Potentials

The zeta potential (ζ) of surface-coated Gd_2_O_3_ NPs dispersed in water provides insight into their surface charge in aqueous media. Negative zeta potentials indicate the presence of negatively charged functional groups such as -OH and -COOH on NP surfaces, whereas positive zeta potentials reflect the presence of positively charged functional groups such as -NH_2_. Zwitterionic ligands such as amino acids typically result in low zeta potential values [100]. High absolute zeta potential values are needed to ensure good colloidal stability [101,102].

For example, Marasini et al. reported polyaspartic acid (PSA)-coated Gd_2_O_3_ NPs as a dual-modal T_1_ and T_2_ MRI CA [63]. Figure 5c shows a highly negative zeta potential of −28.0 mV in aqueous media, consistent with their observed good colloidal stability, evidenced by the absence of NP precipitation following synthesis.

### 3.6. Magnetic Properties

The magnetic properties of surface-coated Gd_2_O_3_ NPs are typically assessed by recording magnetization (M) versus applied magnetic field (H) (M–H curves) and M versus temperature (T) (M–T curves) using either VSM or MPMS. The M–H curves provide saturation magnetization, coercivity, and remanence, while the M–T curves yield the blocking temperature. These curves are measured using 10–20 mg of powdered samples. Notably, M values of surface-coated Gd_2_O_3_ NPs arises predominantly from the spin magnetic moment of Gd^3+^ (s = 7/2), and is largely independent of both ligand type and particle size [21].

**Figure 6:**
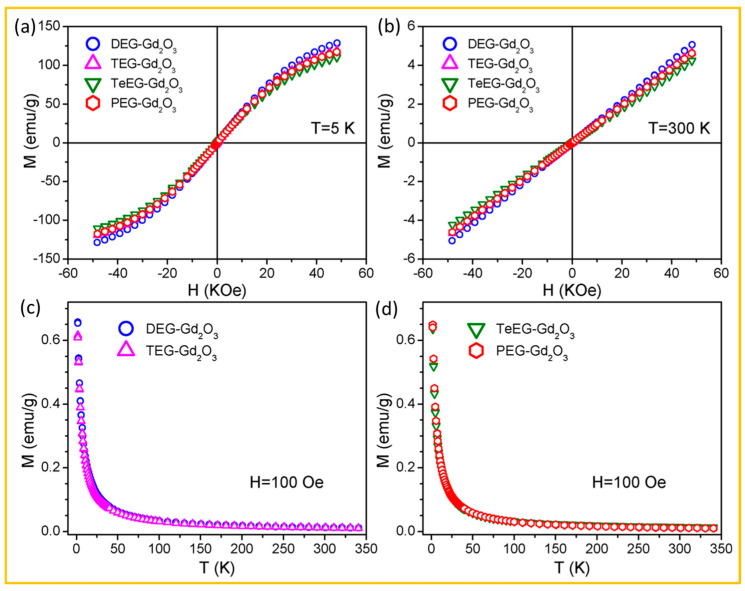
M–H curves of DEG-, TEG-, TeEG-, and PEG-coated Gd_2_O_3_ NPs at (**a**) T = 5 and (**b**) 300 K. M–T curves of (**c**) DEG- and TEG-coated Gd_2_O_3_ NPs, and (**d**) TeEG- and PEG-coated Gd_2_O_3_ NPs at H = 100 Oe (T = 2–340 K) [70].

For example, Guleria et al. measured M–H and M–T curves of various polyol-coated Gd_2_O_3_ NPs [70]. Figure 6a and Figure 6b show the M–H curves of DEG-, TEG-, TeEG-, and PEG-coated Gd_2_O_3_ NPs at 5 and 300 K, respectively. The higher M values at 5 K are attributed to enhanced spin alignment of 4f-electron spins (s = 7/2) at lower temperatures, due to reduced thermal fluctuation. Consistent with the absence of hysteresis in the M–H curves, no magnetic transitions were observed in the M–T curves as depicted in Figure 6c,d, confirming the paramagnetic nature of the Gd_2_O_3_ NPs down to T = 2 K. Moreover, the negligible variation in magnetization among the DEG-, TEG-, TeEG-, and PEG-coated Gd_2_O_3_ NPs confirms that the magnetic moment originates primarily from the paramagnetic Gd_2_O_3_ core, as the surface-coating ligands are nonmagnetic and have minimal influence on the magnetic behavior.

### 3.7. Water Proton Spin Relaxivities (r_1_ and r_2_ Values)

In MRI, T_1_ (longitudinal or spin-lattice) and T_2_ (transverse or spin-spin) relaxation times indicate how quickly the excited proton spins return to their equilibrium state. T_1_ describes the recovery time of the longitudinal (i.e., z-component) magnetization along the main magnetic field. T_2_ describes the decay time of the transverse (i.e., x-y component) magnetization perpendicular to the main magnetic field [103,104].

**Figure 7:**
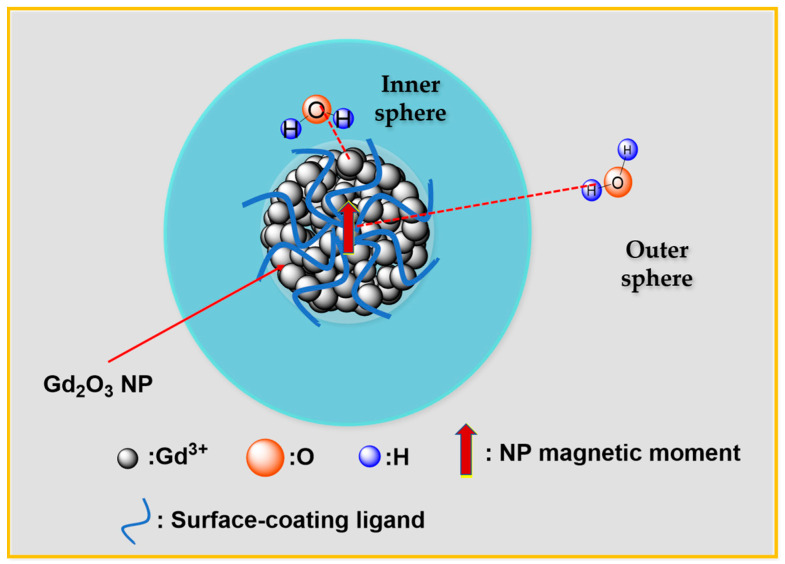
Inner and outer sphere water proton spin relaxation mechanisms. The dotted lines indicate Gd^3+^–water and NP magnetic moment–water interactions. O^2−^ ions were omitted in Gd_2_O_3_ NP.

The positive (T_1_) contrast efficacy increases with increasing r_1_ value while keeping r_2_/r_1_ ratio close to one, whereas negative (T_2_) contrast efficacy increases with increasing r_2_ value with r_2_/r_1_ ratio as large as possible. The r_1_ and r_2_ values are determined by measuring T_1_ and T_2_ water proton spin relaxation times at various Gd concentrations in aqueous solution samples using an MRI scanner. The inverse 1/T_1_ and 1/T_2_ relaxation times are plotted as a function of Gd concentration, and r_1_ and r_2_ values are obtained from the corresponding slopes. The r_2_ value is always greater than r_1_ because T_1_ relaxation always accompanies T_2_ relaxation whereas the opposite does not happen, making r_2_/r_1_ ratio always greater one. Therefore, positive (T_1_) contrast efficacy increases with increasing r_1_ value with r_2_/r_1_ ratio close to one, which are preferred to T_1_ MRI contrast agents. As shown in Figure 7, T_1_ relaxation is primarily induced by the interaction between the Gd^3+^ ion on Gd_2_O_3_ NP surface and water molecule (called inner sphere interaction model), whereas T_2_ relaxation is induced by the interaction between NP magnetic moment and water molecule (called outer sphere interaction model) [104].

**Figure 8:**
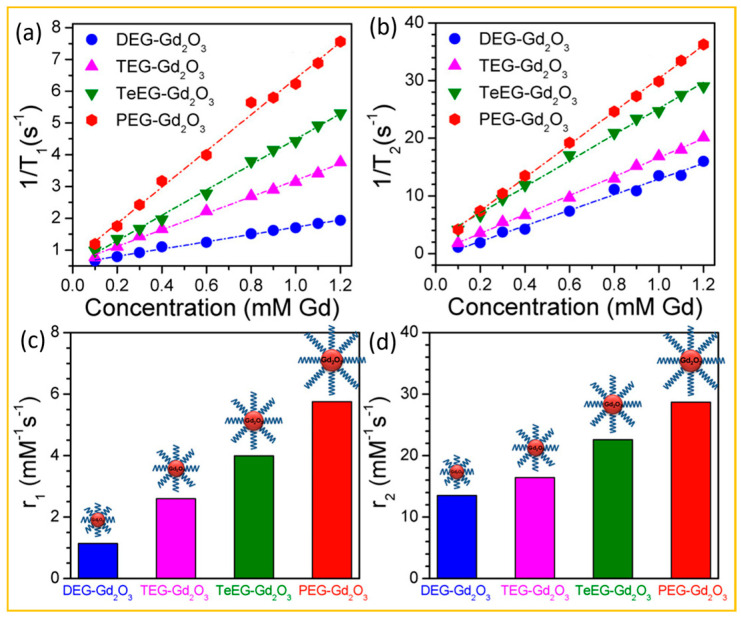
Plots of (**a**) inverse 1/T_1_ and (**b**) 1/T_2_ relaxation times of DEG-, TEG-, TeEG-, and PEG-coated Gd_2_O_3_ NPs dispersed in water as functions of Gd concentration at 3 T. The slopes correspond to r_1_ and r_2_ values, respectively. Effect of glycol chain length on (**c**) r_1_ and (**d**) r_2_ values [70].

For example, Guleria et al. measured the r_1_ and r_2_ values of Gd_2_O_3_ NPs coated with various ligands [70]. Figure 8a and Figure 8b depict the plots of 1/T_1_ and 1/T_2_ relaxation times, respectively, for DEG-, TEG-, TeEG-, and PEG-coated Gd_2_O_3_ NPs dispersed in water as function of Gd concentration. Both r_1_ and r_2_ values increased with increasing glycol chain length, as displayed in Figure 8c and Figure 8d, respectively, suggesting that proton relaxation rates are proportional to the thickness of the hydrophilic coating layer, probably due to enhanced access of outer-sphere water molecules facilitated by the thicker coating.

### 3.8. Toxicity of Surface-Modified Gd_2_O_3_ NPs

Gd_2_O_3_ NPs have demonstrated significant potential as T_1_ MRI CAs due to their considerably higher r_1_ and r_2_ values compared to Gd(III)-chelates [47,97,105,106,107,108,109,110,111,112]. However, their clinical translation is hindered by concerns over toxicity, primarily because free Gd^3+^ ions are toxic and cannot be metabolized by the body [113,114]. Therefore, complete excretion of injected Gd_2_O_3_ NPs, preferably via the renal system, without the release of free Gd^3+^ ions is essential. For clinically approved Gd(III)-chelates, clinical acute and chronic toxicities are well reported [113,114]: acute toxicity includes immediate hypersensitivity allergic-like reactions and physiologic reactions such as coldness, warmth, or pain at the injection site, nausea with or without vomiting, headache, paresthesia, and dizziness and chronic toxicity includes NSF, kidney disease, neurotoxicity, and gadolinium deposition disease such as bone pain and skin and subcutaneous tissue burning pain. However, there exist no clinical studies on acute and chronic toxicity for surface-modified Gd_2_O_3_ NPs. Therefore, we discussed in vitro cellular and in vivo animal toxicity studies reported so far in this section. Notably, free Gd^3+^ ion has an ionic radius comparable to that of Ca^2+^, enabling them to interfere with Ca^2+^-dependent metabolic process and potentially cause adverse effects [115,116]. To mitigate this risk, Gd_2_O_3_ NPs must be coated with hydrophilic and biocompatible ligands such as PAA, PMVEMA, PAAMA, PASA, D-glucuronic acid, CA, dextran, PEG, and PVP [51,58,61,63,64,66,68,73,75,93]. This review addresses both in vitro cellular toxicity and in vivo toxicity (i.e., histological analysis, body weight variation, biodistribution, clearance, and immune response).

Although there is a dose conversion formula for drugs between animals and human [117], the FDA-approved dose for gadolinium-based MRI contrast agents is 0.1 mmol/kg for human as well as animals, except for the liver-specific gadoxetic acid dose (0.025 mmol/kg) [118].

#### 3.8.1. In Vitro Cellular Toxicity

In vitro cellular toxicity studies should be conducted prior to in vivo animal experiments to ensure that the surface-coated Gd_2_O_3_ NPs are nontoxic at the cellular level. Experimental evidence indicates that bare Gd_2_O_3_ NPs are inherently toxic; thus, surface coating with hydrophilic and biocompatible ligands is necessary to reduce cytotoxicity [69,93,119].

Common techniques for assessing cell viability include MTT, WST-1, and CCK-8 assays [120,121]. The MTT assay is a colorimetric method based on the metabolic activity of the cells, whereby mitochondrial dehydrogenases enzymes reduce the tetrazolium salt MTT into water-insoluble formazan crystals. After incubating cells with surface-coated Gd_2_O_3_ NPs (typically for 48 h at 37 °C), the resulting formazan crystals are solubilized using an organic solvent or detergent, and absorbance is measured at 570 nm. The WST-1 assay operates on a similar principle, but uses the tetrazolium salt WST-1, which is converted into a highly water-soluble formazan product by mitochondrial dehydrogenase enzymes; absorbance is measured at 450 nm following incubation with NP samples. The CCK-8 assay employs the water-soluble tetrazolium salt WST-8, which is reduced by viable cells into a water-soluble formazan product that also absorbs at 450 nm. Among these assays, WST-8 has been reported as the most sensitive.

**Figure 9:**
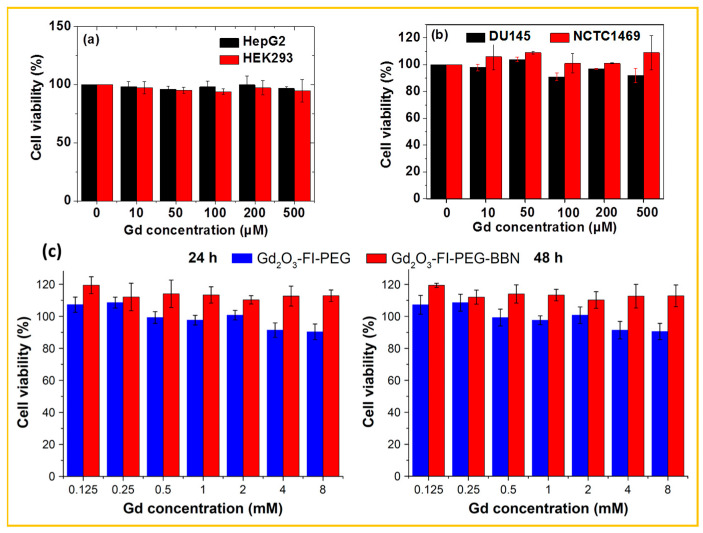
In vitro cell viabilities of (**a**) PAA-Gd_2_O_3_ NPs in HEK293 and HepG2 cell lines after 48 h of incubation [122], (**b**) PAAMA-coated Gd_2_O_3_ NPs in DU145 and NCTC1469 cell lines after 48 h of incubation [61], and (**c**) Gd_2_O_3_-FI-PEG-BBN and Gd_2_O_3_-FI-PEG in PC-3 cell line after 24 h (left) and 48 h (right) of incubation [77].

For example, Ahmad et al. demonstrated the nontoxicity of PAA-coated Gd_2_O_3_ NPs in normal human embryonic kidney (HEK293) and human liver cancer (HepG2) cell lines [120]. As shown in Figure 9a, PAA-coated Gd_2_O_3_ NPs exhibited high cell viability in both cell lines up to a Gd concentration of 500 μM. Figure 9b presents the in vitro cytotoxicity results of PAAMA-coated ultrasmall Gd_2_O_3_ NPs in human prostate cancer (DU145) and normal mouse hepatocyte (NCTC1469) cell lines, showing no toxicity up to 500 µM Gd [61]. Similarly, Cui et al. evaluated the in vitro cytotoxicity of Gd_2_O_3_-FI-PEG and Gd_2_O_3_-FI-PEG-BBN NPs in the prostate cancer (PC)-3 cell line [77]. As shown in Figure 9c, cells treated with various Gd concentrations for both 24 and 48 h, demonstrated good viability for both types of surface-coated NPs. These findings consistently confirm the nontoxicity of surface-coated Gd_2_O_3_ NPs and underscore the importance of hydrophilic and biocompatible ligand surface coatings in mitigating cytotoxicity.

#### 3.8.2. In Vivo Toxicity

##### Histology

Histological analysis enables the investigation of the in vivo toxicity of ligand-coated Gd_2_O_3_ NPs in organs and muscles at the cellular levels. To perform histological studies, mice are intravenously (IV) injected with ligand-coated Gd_2_O_3_ NPs and sacrificed at designated time points after injection to extract relevant organs and muscles. The harvested tissues are sectioned and imaged using an optical microscope, and the results are compared with those of control animals that did not receive NP injections.

**Figure 10:**
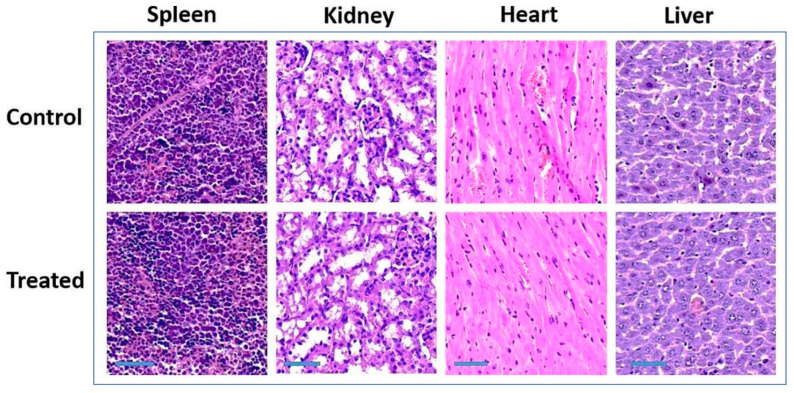
Histological analysis of the liver, kidney, spleen and heart 30 days after IV administration of Gd_2_O_3_@BSA NPs into mice tails (scale bar: 50 μm; number of mice, *N* = 4) [102].

For example, Sun et al. conducted a histological assessment of major organs such as the liver, kidney, spleen, and heart 30 days after IV administration of Gd_2_O_3_@BSA NPs [102]. As shown in Figure 10, no significant alterations or signs of toxicity were observed compared to control tissues, indicating the nontoxicity of Gd_2_O_3_@BSA NPs.

##### Body Weight Change

**Figure 11:**
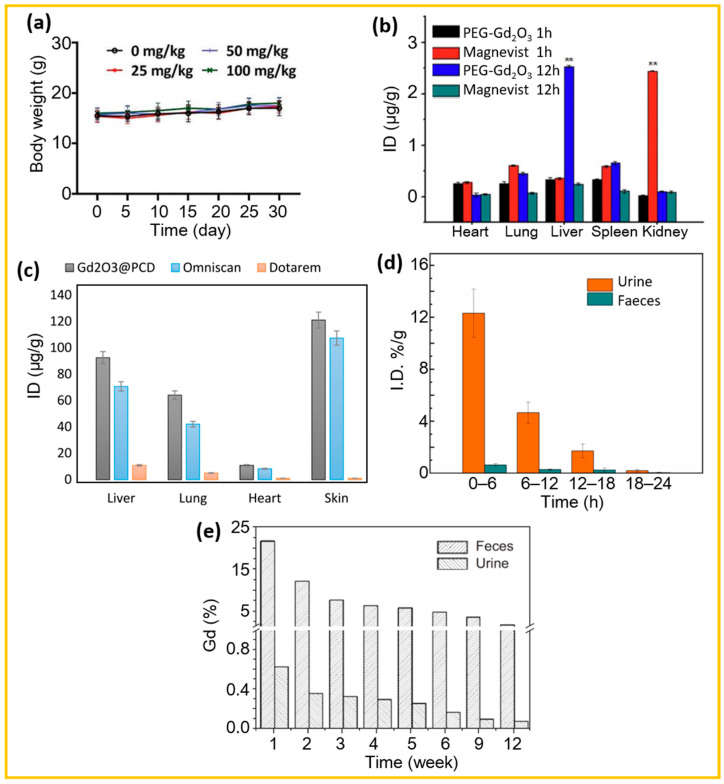
(**a**) Body weight variation over time in mice (*N* = 4) following injection with different doses of Gd_2_O_3_@BSA NPs (25, 50, and 100 mg/kg) compared to a control group (no injection), monitored for 30 days [102]. (**b**) Biodistribution of Magnevist and PEG-Gd_2_O_3_ NPs in the heart, lung, liver, spleen, and kidney at 1 and 12 h after IV injection (15 μmol Gd/kg) (*N* = 3; statistical significance *p* ** < 0.001 compared with the other group in the same organ; ID = percentage of injection dose per gram of organ) [124]. (**c**) Biodistribution of Gd_2_O_3_@PCD NPs, Omniscan, and Dotarem 7 days after tail vein injection (0.2 mmol/kg per week × 6 weeks = total 1.2 mmol Gd/kg for Gd_2_O_3_@PCD NPs; 5.0 mmol/kg per week × 6 weeks = total 30 mmol Gd/kg for Omniscan and Dotarem) (*N* = 5) [125]. (**d**) Time-dependent excretion of ES-GdON-PMA-9 (d = 2.1 nm) via urine and feces after IV injection (5.0 mg Gd/kg) (*N* = 3; I.D.%/g = percentage of injected dose per gram of urine or feces) [128]. (**e**) Excretion profile of Gd_2_O_3_@SiO_2_ NPs (d = 37.29 ± 1.03 nm) through feces and urine up to 12 weeks after mice tail vein injection (15 µmol/kg) (*N* = 3) [129].

Body weight is another important indicator of in vivo toxicity and is measured using a balance before and at regular intervals after IV injection of surface-coated Gd_2_O_3_ NPs into mice tails. In the absence of NP-induced toxicity, body weight should remain stable over time. As shown in Figure 11a, no significant weight loss or gain was observed in mice at any of the tested injection doses (25, 50, and 100 mg/kg) compared to the control group without NP injection, indicating that the Gd_2_O_3_@BSA NPs did not induce toxicity [102].

##### Biodistribution

Biodistribution studies enable the tracking of injected NPs within the body. A solution of Gd-containing NPs is IV injected into mice tails, which are then sacrificed at specific time points after injection to extract organs and muscle for quantification of accumulated Gd concentrations. The biodistribution profile of injected NPs depends on particle size, morphology, and the nature of surface-coating ligands. Ideally, the NPs should be completely excreted from the body via the renal system, similar to Gd(III)-chelates, as free Gd^3+^ ions are toxic and cannot be eliminated through normal metabolic processes [123].

For example, Dai et al. investigated the biodistribution of PEG-Gd_2_O_3_ NPs (hydrodynamic diameter = 30–40 nm) in comparison with commercial Magnetist (Gd-DTPA) as control. They measured Gd accumulation in the heart, lung, liver, spleen, and kidneys at 1 and 12 h after IV injection into mice tails (injection dose = 15 μmol Gd/kg) [124]. As shown in Figure 11b, Magnevist exhibited considerably higher accumulation in the kidneys compared to other organs at 1 h post-injection, indicating rapid renal clearance. Additionally, the Gd concentration in all organs was higher for Magnevist than for PEG-Gd_2_O_3_ NPs, suggesting faster systemic circulation. At 12 h post-injection, Gd levels in all organs for Magnevist were very low, confirming efficient renal excretion. In contrast, PEG-Gd_2_O_3_ NPs showed considerable hepatic accumulation at 12 h, with Gd levels in the liver approximately 7.53 times higher than at 1 h. While the Gd concentration in the heart decreased over time, it increased in the lung, spleen and kidneys, indicating prolonged circulation and predominant hepatic clearance of PEG-Gd_2_O_3_ NPs. As another example, Ashouri et al. investigated the biodistribution of β-cyclodextrin-coated Gd_2_O_3_ (Gd_2_O_3_@PCD) NPs (d < 100 nm, a = 967.6 nm), using Omniscan (Gd-DTPA-BMA) and Dotarem (Gd-DOTA) as commercial controls in renally impaired rats [125]. As shown in Figure 11c, Gd_2_O_3_@PCD NPs exhibited only slightly higher Gd accumulation in all organs compared to Omniscan but considerably higher Gd accumulation than Dotarem, 7 days after tail vein injection (total Gd dose: 1.2 mmol Gd/kg for Gd_2_O_3_@PCD NPs; 30 mmol Gd/kg for Omniscan and Dotarem, administered as 0.2 or 5.0 mmol/kg per week for 6 weeks). These findings indicate that Dotarem was efficiently excreted through the renal system even in renally impaired rats, whereas both Gd_2_O_3_@PCD NPs and Omniscan showed notable organ accumulation, particularly in the skin. Yang et al. investigated the long-term biodistribution of the Gd(OH)_3_ nanorods in mice up to 150 days post-injection [56]. The Gd(OH)_3_ nanorods predominantly accumulated in the liver, spleen, and lungs. They showed that most Gd(OH)_3_ nanorods were rapidly cleared from the circulating blood, with accumulation in these organs increasing initially and then decreasing over time.

##### Clearance

Surface-modified Gd_2_O_3_ NPs should be completely cleared from the body within appropriate time frames after injection owing to their inherent toxicity. Clearance occurs via two primary pathways: renal excretion and hepatobiliary excretion, with the renal excretion being the preferred route. The feasibility of renal excretion primarily depends on particle size and hydrodynamic diameter. Gd_2_O_3_ NPs with diameters smaller than 5 nm and hydrodynamic diameter below 10 nm can be efficiently excreted through the renal system [123,126,127], whereas larger NPs are typically sequestered by macrophages in the liver and spleen and cleared via the hepatobiliary pathway [128,129,130].

For instance, for ultrasmall NPs (d < 5 nm), Wu et al. synthesized polymaleic acid (PMA)-coated extremely small gadolinium oxide NPs (ES-GON-PMA) (d = 2.1 nm) and evaluated their clearance following IV tail injection at a dosage of 5.0 mg/kg [128]. Gd levels were measured in urine and feces over a 24 h period. As shown in Figure 11d, urinary Gd content decreased rapidly over time, while fecal Gd levels remained negligible, indicating predominant renal excretion due to the ultrasmall particle size of ES-GdON-PMA. In contrast, for large NPs (d > 5 nm), Tian et al. investigated the excretion profile of larger Gd_2_O_3_@SiO_2_ NPs (d = 37.29 ± 1.03 nm) after IV administration into mice tails (15 µmol/kg) [129]. As shown in Figure 11e, excretion was monitored weekly in both feces and urine over a period of 12 weeks. The results exhibited considerably higher Gd content in feces than in urine, indicating that the large NPs were primarily cleared via the hepatobiliary pathway due to their insufficient renal clearance.

##### Immunotoxicity

The immunotoxicity assay involves the measurements of (1) ROS in neutrophils from peripheral blood, (2) innate immunity cluster of differentiation (CD) markers such as CD40, CD206, CD11b, and CD80/CD86 in monocytes/macrophages in peripheral blood, (3) adaptive immunity CD markers such as CD69 in lymphocytes from peripheral blood and lymph node, and (4) cytokines including IL-1β, IL-2, and IL-4 in serum [129,131].

**Figure 12:**
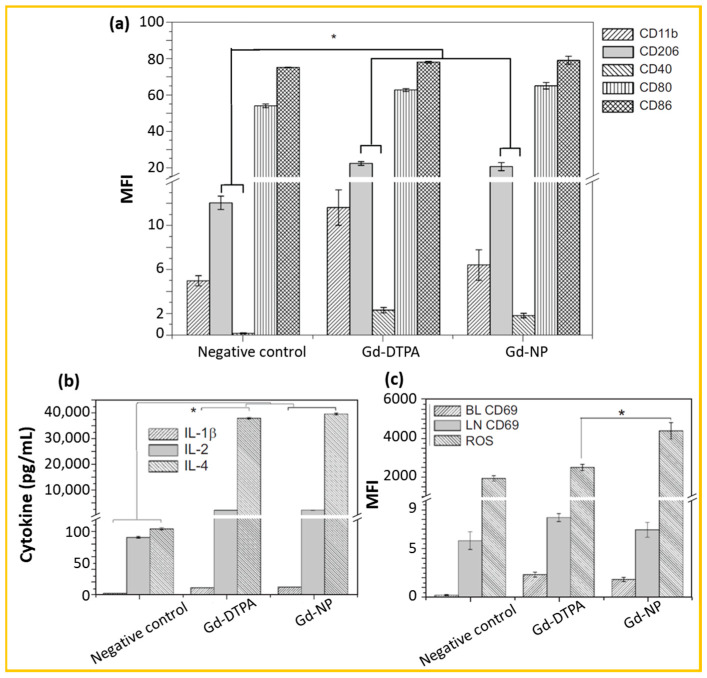
(**a**) Mean fluorescence intensity (MFI) obtained via flow cytometry to evaluate toxicity on innate immune markers (i.e., CD11b, CD206, CD40, CD80, and CD86) in monocytes/macrophages from peripheral blood 48 h after injection of PBS (100 µL, negative control), Gd-DTPA (15 μmol/kg), and Gd-NPs (15 μmol/kg) in BALB/c mice (*N* = 5 per group). (**b**) Serum concentrations of cytokines such as IL-1β, IL-2, and IL-4. (**c**) MFI of the adaptive immune marker CD69 in lymphocytes from peripheral blood and lymph nodes, and ROS levels in neutrophils from peripheral blood. Statistical significance determined by *t*-test (* *p* < 0.05) [129].

For example, Tian et al. evaluated the immunotoxicity of Gd_2_O_3_@SiO_2_ NPs (Gd-NPs; d = 37.29 ± 1.03 nm) by quantifying ROS production, CD maker expression, and cytokine levels in BALB/c mice divided into three groups: PBS (100 µL, negative control), Gd-DTPA (15 µmol/kg), and Gd-NP (15 µmol Gd/kg), with *N* = 5 for each group [129]. As shown in Figure 12a–c, a statistically significant difference was observed between the Gd-NP and PBS groups in the expression levels of CD206, CD40, IL-1β, IL-2, and IL-4. However, no significant difference were found between the Gd-NP and Gd-DTPA groups, except ROS generation. As shown in Figure 12c, the Gd-DTPA and Gd-NP groups exhibited increased ROS production of 29% and 75%, respectively, relative to the PBS group. Although the Gd-NP group showed the highest ROS generation, this result is not critical, as ROS is a nonspecific mediator of immune response, capable of promoting either pathogen clearance or immunosuppression during tissue repair. Therefore, the data suggest that Gd-NPs induce minimal immunotoxicity. Similarly, Zheng et al. investigated the immunotoxicity of Gd_2_O_3_:Eu^3+^ NPs (d = 7.4 nm) in BALB/c mice using PBS and Gd-DTPA as controls [131] and obtained comparable results, further supporting that Gd_2_O_3_:Eu^3+^ NPs exhibit minimal immunotoxicity.

## 4. Factors Affecting r_1_ and r_2_ Values

The r_1_ and r_2_ values of surface-coated Gd_2_O_3_ NPs are key factors determining their performance as MRI CAs [5]. These values are influenced by several factors, such as particle diameter, surface-coating ligands, solution pH, temperature, and applied MR field strength [64,97,132,133,134,135]. Table 3 lists r_1_ and r_2_ values of various surface-coated Gd_2_O_3_ NPs prepared by different methods and measured under diverse conditions, illustrating the dependence of r_1_ and r_2_ values on these factors. This dependence highlights the potential to optimize the efficacy of surface-coated Gd_2_O_3_ NPs as T_1_ MRI CAs through controlled tuning of these variables. Notably, as provided in Table 3, r_1_ values of surface-modified Gd_2_O_3_ NPs are considerably higher than 3–5 s^−1^mM^−1^ [17,38] of clinically approved T_1_ MRI contrast agents, Gd(III)-chelates.

### 4.1. Particle Diameter

Monodispersed ultrasmall Gd_2_O_3_ NPs are preferred for T_1_ MRI applications because a higher proportion of surface Gd^3+^ ions is available to interact with surrounding water molecules, thereby enhancing the r_1_ value. Previous studies have consistently shown that the r_1_ value increases with decreasing particle diameter [80,109,143] and that ultrasmall Gd_2_O_3_ NPs have higher r_1_ values than Gd(III)-chelates, suggesting the existence of an optimal particle size for maximizing r_1_ value. This was demonstrated by Park et al. [64] and Rahman et al. [143]. According to Solomon-Bloembergen-Morgan theory [38,134], the T_1_ relaxation is due to interaction between Gd^3+^ ion and proton spins. As the number of surface Gd^3+^ ions of NPs increases, T_1_ relaxation (or r_1_ value) increases for ultrasmall d < ~2.5 nm [64,143] because majority of Gd^3+^ ions in NPs exist on NP surface due to high surface-to-volume (S/V) ratios, whereas T_1_ relaxation (or r_1_ value) decreases as d increases for d > ~2.5 nm because of low S/V ratios at which majority of Gd^3+^ ions in NPs are internal inactive ones for T_1_ relaxations.

**Figure 13:**
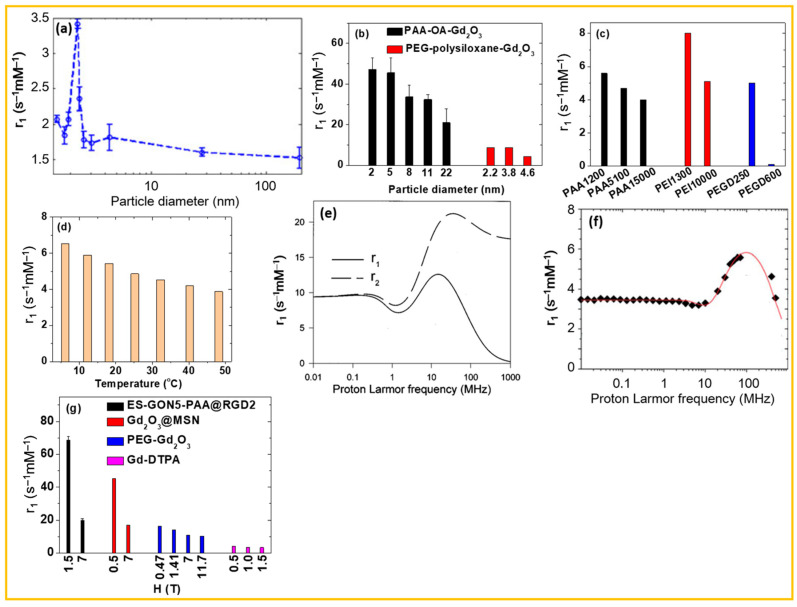
Plot of r_1_ values of (**a**) CA-coated Gd_2_O_3_ NPs as a function of particle diameter (1.5–194.0 nm) at 7 T [143] and (**b**) PAA-octylamine- [80] and PEG-polysiloxane- [109] coated Gd_2_O_3_ NPs as a function of particle diameter at 1.41 and 7 T, respectively. (**c**) Plot of r_1_ values of various ligand-coated Gd_2_O_3_ NPs as a function of ligand size at 1.5 T [71,75,97]. (**d**) Plot of r_1_ values of Gd(OH)_3_ NPs as a function of temperature at 1.41 T [144]. (**e**) Theoretical NMRD profile of superparamagnetic NPs (d = 5 nm) [133]. (**f**) NMRD profile of CA-coated GdF_3_ NPs (d = 2.2–2.3 nm) [135]. (**g**) Plots of r_1_ values of various ligand-coated Gd_2_O_3_ NPs and Gd(III)-DTPA as a function of H [73,107,140,146].

Rahman et al. reported that the r_1_ value of CA-coated Gd_2_O_3_ NPs increased as particle size decreased below 3.0 nm at 7 T, reaching a maximum value at approximately 2.3 nm; further size reduction led to a decline in r_1_ value (Figure 13a) [143]. Park et al. roughly estimated the optimal particle size to lie between 1.1 and 2.5 nm [64]. In another study, the r_1_ values of PAA-octylamine-coated Gd_2_O_3_ NPs (d = 2, 5, 8, 11, and 22 nm) [80] and PEG-polysiloxane-coated Gd_2_O_3_ NPs (d = 2.2, 3.8, and 4.6 nm) [109] decreased with increasing particle diameter at 1.41 and 7 T, respectively (Figure 13b). This trend likely results from particle diameters exceeding the optimal range. Conversely, the r_2_ value, which is influenced by the NP magnetic moment, tends to increase with particle size due to the corresponding increase in magnetic moment per NP [64].

### 4.2. Surface-Coating Ligands

Surface coating is another critical factor influencing the r_1_ and r_2_ values of Gd_2_O_3_ NPs [71,75,97]. Appropriate selection of coating ligands can enhance the accessibility of water molecules near the Gd_2_O_3_ NP surface, thereby increasing the r_1_ and r_2_ values.

For example, Tegafaw et al. investigated the relaxometric properties of Gd_2_O_3_ NPs coated with various ligands, including small diacids with hydrophobic chains (succinic acid, glutaric acid, and terephthalic acid) and large PEIs (PEI-1300 and PEI-10,000) with hydrophilic chains [71]. As shown in Figure 13c, both r_1_ and r_2_ values decreased with increasing ligand size. This indicates that small, hydrophilic ligands promote closer interaction between water molecules and the NP surface, thereby enhancing r_1_ and r_2_ values. Similarly, Miao et al. reported that the r_1_ and r_2_ values of ultrasmall Gd_2_O_3_ NPs decreased as the molecular weight of PAA (M_w_  =  1200, 5100, 15,000 Da) increased (Figure 13c) [75]. Kim et al. also observed a reduction in r_1_ and r_2_ values as the molecular weight of PEGD (M_w_  =  250 and 600 Da) increased (Figure 13c) [97]. These results indicate that smaller, hydrophilic ligands are preferable for achieving higher r_1_ and r_2_ values.

### 4.3. Solution pH

The solution pH influences the surface charge and colloidal stability of surface-coated Gd_2_O_3_ NPs, thereby affecting their r_1_ and r_2_ values. pH can be adjusted by adding acid or base. For example, in the case of PAA-coated Gd_2_O_3_ NPs, the carboxyl groups on PAA ligands become protonated under acidic conditions, potentially leading to NP aggregation. Aggregation increases particle size and reduces the surface-to-volume ratios, which may limit water exchange between bulk water and the Gd_2_O_3_ NP surface, thereby decreasing the r_1_ value. Conversely, under basic conditions, the carboxyl groups are deprotonated, resulting in negatively charged surfaces that promote electrostatic repulsion between NPs. This enhanced repulsion improves colloidal stability and increases water accessibility to the NP surface, potentially enhancing r_1_ values. In contrast, the r_2_ value, which increases with the NP magnetic moment [64], may rise with NP aggregation due to the increase in the magnetic moment per NP. However, excessive aggregation can still compromise colloidal stability, which may in turn limit this effect.

### 4.4. Temperature

The r_1_ and r_2_ values are also influenced by temperature, as key parameters such as the diffusion coefficient (D) and rotational correlation time (τ_R_) of water molecules and CAs are temperature-dependent [133,134,144,145]. As temperature increases, D increases and τ_R_ decreases, thereby shortening the interaction time between water proton spins and the NPs. This reduction in interaction time leads to decreased r_1_ and r_2_ values. For example, Goussuin et al. demonstrated that the r_1_ and r_2_ values of Gd(OH)_3_ NPs decreased with increasing temperature at 1.41 T, as shown in Figure 13d [144].

### 4.5. Applied Magnetic Field Strength (H)

Roch et al. theoretically investigated the r_1_ and r_2_ values of superparamagnetic NPs (d = 5 nm) as a function of the applied magnetic field strength (H) or proton Larmor frequency [133]. As shown in Figure 13e, three distinct regions were identified: Region 1, where r_1_ and r_2_ values remain nearly constant at H < 1 MHz; Region 2, where r_1_ and r_2_ values increase with H between 1 and 10 MHz; and Region 3, where r_1_ and r_2_ values decrease at H > 10 MHz (the clinical region).

For example, Carniato et al. synthesized CA-coated GdF_3_ NPs (d = 2.2–2.3 nm) and observed three regions in the r_1_ nuclear magnetic resonance dispersion (NMRD) profile, as shown in Figure 13f [135], in agreement with theoretical prediction [133]. Additional experimental r_1_ value plots as a function of H in Region 3 are displayed in Figure 13g, demonstrating that the r_1_ values of various ligand-coated Gd_2_O_3_ NPs and Gd(III)-chelates decrease with increasing H in this region [73,107,140,146]. Therefore, the proton signal enhancement by T_1_ MRI CAs diminishes with increasing H in the clinical region. However, due to enhanced proton spin alignment along H at higher field strengths, resulting in a larger proton spin population difference, the net proton signal enhancement at high H remains considerably greater than that predicted solely from the T_1_ MRI CA contribution.

## 5. In Vivo MRI Applications

### 5.1. Imaging in Normal Mice

**Figure 14:**
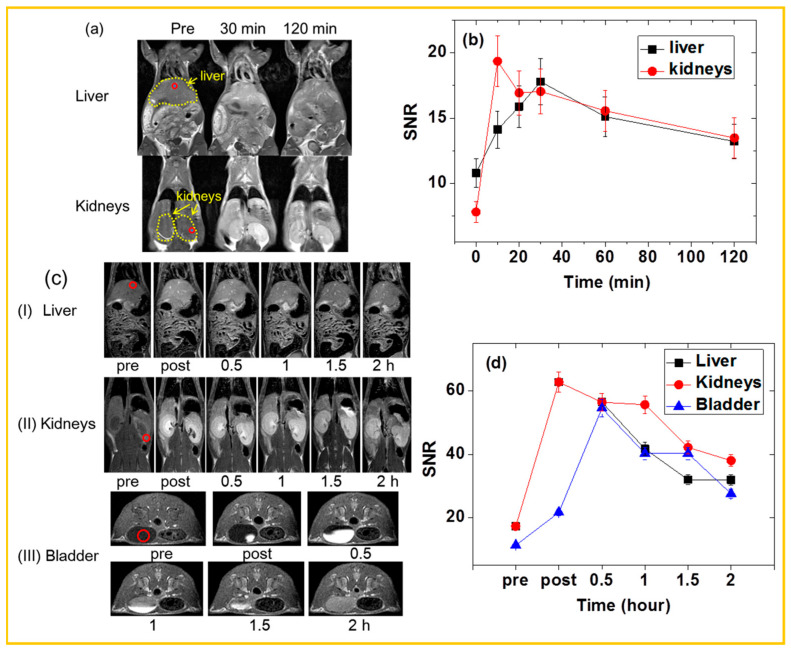
(**a**) In vivo T_1_ MR images of a mouse at 3 T before (“Pre”) and after IV administration of the solution sample via the mouse tail. Small circles indicate ROIs, and dotted circles indicate the liver and kidneys (administration dose = ~0.1 mmol Gd/kg) and (**b**) SNR plots of the ROIs in the liver and kidneys as a function of time [58]. (**c**) In vivo coronal and axial T_1_ MR images of the (I) liver, (II) kidneys, and (III) bladder of mice before (labeled as pre) and after IV injection of Gd_2_O_3_@C NPs (administration dose = ~0.1 mmol Gd/kg) and (**d**) SNR-ROI plots as a function of time [69]. The red circles in MR images indicate ROIs.

The FDA-approved dose for gadolinium-based MRI contrast agents is 0.1 mmol/kg for human as well as animals, except for the liver-specific gadoxetic acid dose (0.025 mmol/kg) [118]. Numerous in vivo studies have reported the use of surface-coated Gd_2_O_3_ NPs as T_1_ MRI CAs, demonstrating their strong potential as T_1_ MRI CAs. For example, Ahmad et al. investigated PMVEMA-coated ultrasmall Gd_2_O_3_ NPs (d = 1.9 nm) as a T_1_ MRI CA [58]. Following IV administration of the aqueous NP solution into mice tails, positive (i.e., bright) contrast enhancement was observed in the liver and kidneys, as shown in Figure 14a. Signal-to-noise ratios (SNRs) of regions of interest (ROIs) in these organs initially increased, reached maxima, and then decreased over time, indicating accumulation followed by excretion of the NPs, predominantly through the renal pathway, thus confirming their role as T_1_ MRI CAs (Figure 14b). In another example, Yue et al. reported carbon-coated Gd_2_O_3_ (Gd_2_O_3_@C) NPs (d = 3.1 nm) as a T_1_ MRI CA [69]. Upon IV administration into mice tails, the NPs produced positive contrast enhancement in the liver, kidneys, and bladder (Figure 14c). The SNR-ROIs in these organs, plotted as a function of time (Figure 14d), showed that the contrast enhancements peaked between the point of administration and 30 min post-injection, and subsequently declined. These results further confirm the function of the NPs as a T_1_ MRI CA.

### 5.2. Imaging in Cancer Model Mice

Owing to the EPR effects, NPs can accumulate more readily in cancer cells than in normal cells (called passive targeting) [147]. Furthermore, the accumulation of NPs in cancer cells can be considerably enhanced by conjugating cancer-targeting ligands onto the NP surface (called active targeting).

**Figure 15:**
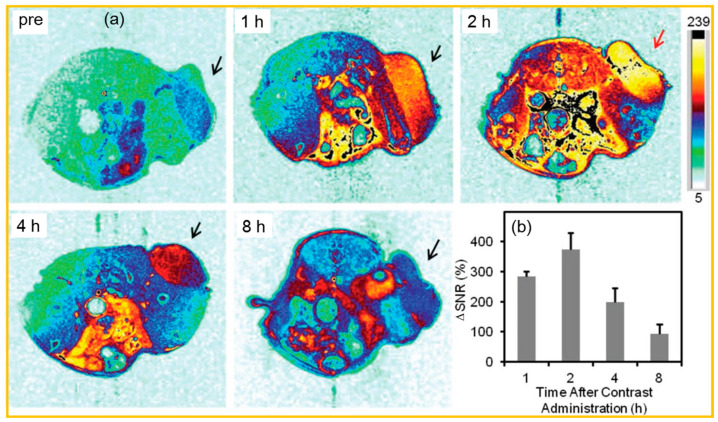
(**a**) In vivo axial T_1_ MR images of U87MG tumor-bearing nude mice acquired at 1.5 T after IV injection of ES-GON5-PAA@RGD2 NPs (arrows indicate the tumor). (**b**) ΔSNR in the tumor at various time points. The injection Gd dosage was 5.0 mgkg^−1^ [107].

Dai et al. showed that PEG-Gd_2_O_3_ NPs (r_1_ = 29.0 s^−1^ mM^−1^) displayed stronger contrast enhancement of the tumor in in vivo MR images than Magnevist (r_1_ = 4.2 s^−1^ mM^−1^) at the same amount of Gd injection, demonstrating the superiority of PEG-Gd_2_O_3_ NPs to commercial molecular contrast agent Magnevist [124]. Shen et al. evaluated T_1_ MR images of U87MG tumor model nude mice using exceedingly small (ES), RGD2-conjugated PAA-coated Gd_2_O_3_ (simply referred to as ES-GON5-PAA@RGD2) NPs [107]. As shown in Figure 15a, the tumor region exhibited a very weak MRI signal prior to IV injection. Following injection, signal intensity increased markedly, peaking at 2 h post-administration. Quantitative analysis in Figure 15b revealed a maximum ΔSNR [=100% × (SNRpost − SNRpre)/SNRpre] of 372 ± 56%, which is considerably higher than the ΔSNR of <80% of typically observed value with conventional Gd(III)-chelates. This remarkably enhanced tumor ΔSNR can be attributed to the combined effects of the high relaxivity value of the NPs (r_1_ = 68.7 ± 2.3 mM^–1^s^–1^, r_2_/r_1_ = 1.03 ± 0.03 at 1.5 T), the active targeting capability of RGD2 toward integrin αvβ3-overexpressed on tumor cells, and the EPR effect.

## 6. Conclusions and Future Outlook

Considerable research progress has been made on surface-coated Gd_2_O_3_ NPs as promising T_1_ MRI CAs. However, no comprehensive review on this subject has been reported to data. Therefore, a thorough overview addressing current status and future perspectives of surface-coated Gd_2_O_3_ NPs as T_1_ MRI CAs is highly warranted. Such a review would substantially advance research in this field and assist researchers in understanding the current state of the art and identifying future research directions.

This review has covered synthesis methods (i.e., polyol, DMSO, thermal decomposition, and hydrothermal techniques); characterization techniques (i.e., TEM, DLS, XRD, FTIR, TGA, zeta potential, VSM, and MRI); in vitro cellular and in vivo toxicity assessments (i.e., histology, biodistribution, body weight change, clearance, and immunotoxicity); and in vivo MRI applications of surface-modified Gd_2_O_3_ NPs. In addition, the influence of various factors (i.e., particle diameter, surface-coating ligands, solution pH, temperature, and applied MR field) on performance parameters of MRI CAs (i.e., r_1_ and r_2_ values) was discussed, as this information is critical for the rational design of optimized Gd_2_O_3_ NPs for specific applications. By comparing the strengths and limitations of each synthesis method, researchers can select the most appropriate approach for their intended application. The characterization protocols described herein may also serve as standard procedures for studies. Although previous studies have demonstrated the strong potential of surface-modified Gd_2_O_3_ NPs as T_1_ MRI CAs, further development of advanced formulations that fully meet in vivo toxicity criteria is essential for clinical translation. For instance, nontoxic, renally excretable formulations that prevent the release of free Gd^3+^ ions should be pursued.

While remarkable progress has been achieved in surface-modified Gd_2_O_3_ NPs, successful translation to clinical trial phases demands standardized methodologies. Future research should focus on conducting comprehensive toxicity assessments, including acute, sub-chronic, and chronic studies aligned with Food and Drug Administration (FDA) and European Medicines Agency (EMA) guidelines [148,149]. Standardized protocols for evaluating renal clearance and nephrotoxicity are also needed for clinical trial phases. Additionally, scalable manufacturing processes compliant with Good Manufacturing Practices (GMPs) are necessary for commercialization. GMP-compliant scale-up strategies involve a systematic approach to increasing production volume while maintaining product quality and regulatory compliance. Key aspects include facility design, process validation, quality control, supply chain management, and workforce training; all tailored to meet GMP requirements.

The surfaced-modified Gd_2_O_3_ NPs exhibited higher r_1_ values compared to surfaced-modified Mn and Fe-based NPs owing to the higher electron spin magnetic moment of Gd^3+^ (s = 7/2) compared to Mn^2+^ (s = 5/2) and Fe^3+^ (s = 5/2). The only disadvantage of surfaced-modified Gd_2_O_3_ NPs compared to surfaced-modified Mn- and Fe-based NPs is their higher potential toxicity. Therefore, advancement in biocompatible ligand coatings is critical to ensure the safety profile of surfaced-modified Gd_2_O_3_ NPs. Several studies assessed colloidal stability of surface-modified lanthanide oxide NPs in water, phosphate-buffered saline (PBS), or serum-containing media [51,69,150], demonstrating good colloidal stability overtime and thus, good coating stability.

Over the past decade, extensive efforts have been devoted to developing new T_1_ MRI CAs as alternatives to Gd(III)-chelates, which suffer from low sensitivity due to their low r_1_ values, as well as non-specificity and short imaging windows resulting from rapid blood clearance. These limitations often necessitate the administration of large doses. Surface-modified Gd_2_O_3_ NPs have emerged as promising alternatives, offering improved performance by overcoming the shortcomings of Gd(III)-chelates. Moreover, surface-modified Gd_2_O_3_ NPs can be easily conjugated with cancer-targeting ligands and therapeutic drugs, enabling targeted theranostic applications. They can be further functionalized via integration with other imaging agents such as FI, CT, PET, and SPECT agents to promote their application as multimodal imaging agents. While many of these advantages have been demonstrated in in vivo animal studies, considerable challenges remain in translating these systems into clinical practice. As discussed above, the development of advanced formulations and comprehensive in vivo toxicity evaluations are critical next steps. Achieving clinical translation will require sustained interdisciplinary research efforts.

## Figures and Tables

**Table 1:** Summary of synthesis methods with associated advantages and disadvantages.

Method	Solvent	Advantage	Disadvantage	Ref.
Polyol	Polyol such as ethylene glycol, diethylene glycol, triethylene glycol, polyethylene glycol	– surface modification in one-pot synthesis – ultrasmall NPs	– small-scale synthesis – poor crystallinity	[51,52]
DMSO	DMSO	– low temperature synthesis (<60 °C) – surface modification in one-pot synthesis – ultrasmall NPs	– small-scale synthesis – poor crystallinity	[53]
Thermal decomposition	High boiling point organic solvent such as oleic acid, oleylamine	– high crystallinity. – monodisperse NPs with particle size control	– expensive (organic solvent, inert gas) – post synthesis surface modification – solvent waste – small-scale synthesis	[55]
Hydrothermal	Water	– environmentally friendly – large-scale synthesis	– requires autoclave	[56]

**Table 2:** Various characterization techniques and information.

Characterization Technique	Information
TEM	Particle size, morphology
ICP-AES	Gd concentration in aqueous solution sample
XRD	Crystal structure
DLS	Hydrodynamic diameter
FT-IR	Surface coating
TGA	Surface-coating amount
Zeta potential	Surface charge
VSM, MPMS	Magnetic properties
MRI	r_1_, r_2_ values
MTT, CCK-8, WST-1	in vitro cellular toxicity

**Table 3:** r_1_ and r_2_ values of surface-coated Gd_2_O_3_ NPs under various conditions, including coating ligands, particle size, hydrodynamic diameter, applied magnetic field, and temperature.

NP	Synthesis Method	Ligand	Size (nm)		r_1_ (s^−1^mM^−1^)	r_2_ (s^−1^mM^−1^)	H (tesla)	T (°C)	Ref.
			TEM	DLS					
Gd_2_O_3_	polyol	Succinic acid	1.3	4.11	12.5	15.4	1.5	22	[71]
		Glutaric acid	1.3	4.15	13	13.2			
		Terephthalic acid	1.3	4.19	11.6	14.4			
		PEI-1300	1.3	12.71	8	9.1			
		PEI-10,000	1.3	13.87	5.1	7.6			
		D-glucuronic acid	2.4		4.25	27.11			
		PAA-1200	2	10.3	5.6	36.6	1.5	22	[75]
		PAA-5100	2	11.1	4.7	31			
		PAA-15,000	2	11.3	4	29.2			
		PEGD-600	0.9		14.18	19.15	1.5	22	[136]
		D-glucuronic acid	1		12.56	12.95			
		Lactobionic acid	0.9		11.57	13.38			
		D-glucuronic acid	1	4	9.9	10.5	1.5	22	[64]
		DEG		17 ± 2	2.1	2.8	1.5	25	[137]
		PEG-phosphate (reaction in water)		152 ± 20	10	14.2			
		PEG-phosphate (reaction in ethanol)		68 ± 11	11.4	14.5			
		PEG-polysiloxane	2.2	3.3	8.8	11.4	7	25	[109]
			3.8	5.2	8.8	28.8			
			4.6	8.9	4.4	28.9			
		D-glucuronic acid	2.4		4.25	27.11	1.5	22	[138]
		Fluorescein-polyethyleneimine	3.92	7.5	6.76	20.27	1.5	22	[139]
		PEG	1.3	9.1	16.2	17.7	0.47		[73]
					14.2	17.2	1.41		
					10.9	15.9	7		
					10.4	17.2	11.7		
		MSN	2.3	62.25	45.08	48.81	0.5		[140]
					16.95		7		
		D-Glucuronic acid	1.3	6.2	24 ± 2	60 ± 5	1.5	22	[97]
		PEGD-250		9.7	5 ± 1	55 ± 2			
		PEGD-600		12.1	0.1 ± 0.1	10 ± 1			
		3,5-Diiodo-L-tyrosine	2		9.24	38.27	1.5		[141]
		Carbon	3.1	18.9	16.26	24.12	1.5	22	[69]
		PAA5100	2	6.3	31	37.4	1.5	22	[51]
		Polyaspartic acid	2	12.7	19.1	53.7	3	22	[63]
		Apoferritin-D-Glucuronic acid	1.9	29.1	8.7	15.7	1.5	22	[142]
		PVP	2.5		10.28	14.47	3		[68]
		DEG	13		1.14	13.5	3		[70]
		TEG	16		2.6	16.4			
		TeEG	19		3.99	22.6			
		PEG	21		5.75	28.7			
		Dextran	1.5	12.4	12.2	29.3	1.5	22	[66]
		Poly(acrylic acid-co-maleic acid) (PAAMA)	1.8	9	40.6	63.4	3	22	[61]
		Poly(methyl vinyl ether-alt-maleic acid) (PMVEMA)	1.9	19.8	36.2	74	3	22	[58]
		PEG	<3	2.8	9.4	13.4	1.5	19.5	[72]
		DEG	<3		6.4	15.2			
		PEG	<40		0.1	7.6			
Gd_2_O_3_	DMSO		4.7	5–10	6.9	7.9	1.41		[53]
		5(6)-carboxyfluorescein-polyethylene glycol-bombesin	52.3	90.6	4.23		3		[77]
		TEG based carboxylate ligand	4	13	6.4	9.1	1.41		[76]
Gd_2_O_3_	Thermal decomposition	PVP	2.9	15.7	12.123	33.184	7	36	[78]
		*N*-Dodecyl-PEI-PEG	9.1	95	14.13		9.4		[55]
		PAA-OA	2	27.8	47.2	82.4	1.41		[80]
			5	28.1	45.6	75.1			
			8	31.2	33.7	70.0			
			11	33.0	32.4	67.3			
			22	47.1	21.1	32.4			
		poly (maleic anhydride-alt-1-octadecene) polymer	10 × 1.1	32	16.95		1.5		[81]
		CTX-PEG-TETT	9.09		8.41		7		[79]
Gd_2_O_3_	Hydrothermal	Hyaluronic acid	105		6		3		[46]
		CA	100 ± 20	233.7 ± 10	1.8	5.3	1.41	40	[93]

## Data Availability

The data presented in this study are contained within the article.
